# *Mycobacterium tuberculosis* Genotype and Case Notification Rates, Rural Vietnam, 2003–2006

**DOI:** 10.3201/eid1510.090170

**Published:** 2009-10

**Authors:** Tran N. Buu, Mai N.T. Huyen, Nguyen N.T. Lan, Hoang T. Quy, Nguyen V. Hen, Matteo Zignol, Martien W. Borgdorff, Dick van Soolingen, Frank G.J. Cobelens

**Affiliations:** Pham Ngoc Thach Tuberculosis and Lung Disease Hospital, Ho Chi Minh City, Vietnam (T.N. Buu, M.N.T. Huyen, N.T.N. Lan, H.T. Quy); Tien Giang Provincial Tuberculosis and Lung Disease Hospital, My Tho, Vietnam (N.V. Hen); World Health Organization, Geneva, Switzerland (M. Zignol); KNCV Tuberculosis Foundation, The Hague, the Netherlands (M.W. Borgdorff, F.G.J. Cobelens); Academic Medical Centre, Amsterdam, the Netherlands (M.W. Borgdorff, F.G.J. Cobelens); National Institute of Public Health and the Environment, Bilthoven, the Netherlands (D. van Soolingen)

**Keywords:** Tuberculosis and other mycobacteria, Mycobacterium tuberculosis, bacteria, genotype, trends, Vietnam, research

## Abstract

Tuberculosis case notification rates (CNRs) for young adults in Vietnam are increasing. To determine whether this finding could reflect emergence of *Mycobacterium tuberculosis* Beijing genotype, we studied all new sputum smear–positive pulmonary tuberculosis patients registered for treatment in 3 rural districts in Vietnam during 2003–2006. Beijing strain infections were more frequent in younger patients (15–24 years of age, 53%) than in older patients (31%; p<0.001). The increase in CNRs for youngest patients was larger for disease caused by the Beijing genotype than by other genotypes, but the difference was not significant. For patients 15–24 years of age, 85% of fluctuations in CNRs between years was caused by fluctuations in Beijing genotype infections compared with 53% and 23% in the groups 25–64 and >65 years of age, respectively (p<0.001). These findings suggest that young adults may be responsible for introducing Beijing strains into rural Vietnam.

## Abstract 2

Young adults may be introducing Beijing strains.

One third of the world’s population is infected with *Mycobacterium tuberculosis*, and ≈9 million tuberculosis (TB) cases were diagnosed worldwide in 2006 (*1*). Introduced in the early 1990s, the directly observed treatment, short-course (DOTS) strategy is an essential component of the Global Stop TB Strategy and regarded as a highly cost-effective method for controlling the TB epidemic (*2*). In addition, the DOTS strategy has resulted in decreased numbers of TB cases in Peru, parts of the People’s Republic of China, India, and Indonesia (*3**–**6*) a few years after those countries met the goals of the World Health Organization (WHO), which are to detect >70% and cure >85% of smear-positive TB cases (*7*).

Conversely, the DOTS strategy has had a limited effect (no decrease in numbers of TB cases) in other regions, such as the former Soviet Union and sub-Saharan Africa (*3**,**8*). In Vietnam, TB case notification rates (CNRs) have not decreased since 1997 when the National TB Control Program reached WHO goals (*1**,**9*). This absence of a stable rate decrease reflects a decrease in TB CNRs among middle-age persons, primarily women, which is compensated for by an increase in CNRs in young adults, primarily men (*10*). Several explanations for this phenomenon have been proposed, including the emerging HIV epidemic (*11*), rapid urbanization (*12*), and emergence of the *M*. *tuberculosis* Beijing genotype (*13*). Studies worldwide indicated that the Beijing genotype is widespread and associated with drug resistance (*14**–**20*). In Vietnam, a study of isolates from patients located mainly in Ho Chi Minh City showed that the Beijing genotype accounted for 55% of the *M*. *tuberculosis* isolates and was associated with young age and drug resistance (*21*). Another study in Ho Chi Minh City found that this genotype was more frequent among patients with treatment failure or relapse (*22*). Therefore, emergence of the Beijing genotype, or a higher rate of recurrence of Beijing genotype cases, could explain part of the increase in TB rates among young adults. However, these studies were conducted in large urban areas where rapid urbanization and internal immigration may have confounded these associations. Therefore, we assessed, in a population-based study, the role of the Beijing genotype in the TB epidemic in a rural setting in Vietnam. We studied trends in CNRs of new smear-positive TB cases caused by specific genotypes over time by age and sex, and age-specific variations in genotype distribution over time.

## Methods

The study was conducted at Pham Ngoc Thach Tuberculosis and Lung Disease Hospital, Ho Chi Minh City, Vietnam, and Tien Giang Provincial Tuberculosis and Lung Disease Hospital, My Tho, Vietnam. The study area consisted of 3 adjacent rural districts in Tien Gang Province, situated in the Mekong River Delta in southern Vietnam. These 3 districts have implemented DOTS strategies since 1994. Each district has a district TB unit that examines sputum smears and treats ambulatory patients with smear-positive results according to the DOTS strategy. HIV testing of TB patients is performed only when HIV infection is suspected on the basis of clinical signs. Details of the study area have been described elsewhere (*13*).

Eligible for inclusion were all patients >15 years of age who were residents in the study area and who had registered for treatment of smear-positive pulmonary TB from January 1, 2003, through December 31, 2006, at the participating district TB units or at the provincial TB hospital, and had started treatment for TB <2 weeks earlier. Smear-positive TB was diagnosed by microscopic examination of >2 Ziehl-Neelsen–stained sputum smears following international guidelines (*23**,**24*). Eligible patients were included in the study after they provided written informed consent. Scientific and ethical clearance was obtained from the Ethical Health Committee of the Ho Chi Minh City Council. For technical reasons, in 1 of 3 districts, data collection did not start until 2004.

### Data Collection and Laboratory Methods

Included patients were asked to submit 2 pretreatment sputum specimens for *Mycobacterium* culture. Specimens were refrigerated and transported to the Mycobacterial Reference Laboratory in Ho Chi Minh City within 72 hours. At the reference laboratory, sputum specimens were decontaminated and liquefied with 1% n-acetyl-l-cysteine, 2% NaOH, placed on modified Ogawa medium, and incubated at 37°C (*23*). Cultures were examined for growth after 1, 2, 4, 6, and 8 weeks of incubation; cultures with no growth after 8 weeks were reported as negative. *M*. *tuberculosis* was identified by using the niacin and nitrate tests, and isolates were genotyped by spoligotyping by using a standardized method (*25*).

Notification data for new patients with smear-positive results, by age and sex, during 1997–2006 were obtained from routine reports of the district TB units in the study site. Sex- and age-specific population denominators were interpolated and extrapolated from 1999 and 2004 census data; standard exponential population growth was assumed.

### Definitions

**Figure 1:**
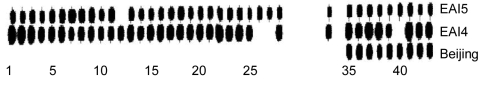
Typical spacer patterns of the *Mycobacterium tuberculosis* spoligotypes most frequently isolated from patients with smear-positive pulmonary tuberculosis, Vietnam, 2003–2006. EAI5 and EAI4 are East African–Indian genotypes.

A new case of new smear-positive TB was defined as a case in a patient who had never had treatment for TB or who had taken drugs for treatment of TB for <1 month (*7*). The Beijing genotype was defined as any isolate without direct repeat spacers 1–34 and with >3 spacers 35–43 by spoligotyping (*26*). Other genotypes were defined as described by Brudey et al. (*27*), including the East-African-Indian Vietnam genotype (EAI-VNM), which belongs to the EAI genotype family of *M*. *tuberculosis* and is considered the most common genotype in Vietnam (*21**,**27*) (Figure 1).

### Data Management and Statistical Analyses

Data were entered into EpiInfo version 6.04 (Centers for Disease Control and Prevention, Atlanta, GA, USA). A 20% random sample was double-entered and discrepancies were checked against raw data. Discrepancies were found in <0.5% of the records and <0.1% of the entries. Analyses were performed by using Stata version 8 (StataCorp LP, College Station TX, USA) and Excel 2003 (Microsoft, Redmond, WA, USA). Patients with negative cultures or cultures that grew nontuberculous mycobacteria were excluded from the analyses.

We used the χ^2^ test for comparison of proportions. Time trends were assessed by using the Cuzick nonparametric test for trends (*28*). To assess overall trends of CNRs, age- and sex-specific CNRs were standardized by direct standardization using the 1999 census population as the reference and plotted against time. Exponential trend lines were fitted by using the least-squares method. We calculated trends of CNRs for 2003–2006 by sex, age group, and genotype by using Poisson regression. Trends over time by genotype and age group were assessed by testing for interactions between these variables and the variable year in Poisson regression models and by using the likelihood ratio χ^2^ test for significance testing.

To analyze variation in genotype distribution over time, we first calculated the absolute differences in CNRs between subsequent years and the proportion of these absolute differences for each genotype. Thereafter, averages of these proportions were calculated; to adjust for differences in changes over time, we weighted these averages by absolute variation per year of summed variation for all years. Significance testing was conducted for numbers of cases (i.e., disregarding the population denominator) by χ^2^ test comparing genotype against combined strata of year and age group. All tests were conducted at the 5% significance level.

## Results

**Figure 2:**
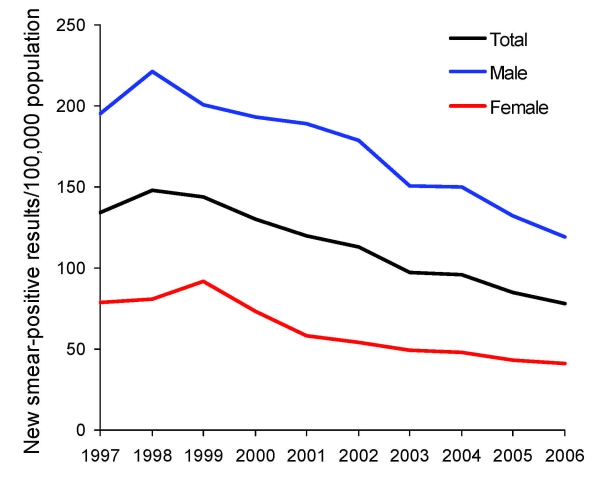
Trends in case notification rates for patients with new smear-positive tuberculosis, by sex, Vietnam, 1997–2006. The annual percentage changes were –4.3% for male patients, –7.7% for female patients, and –5.1% for all persons.

**Figure 3:**
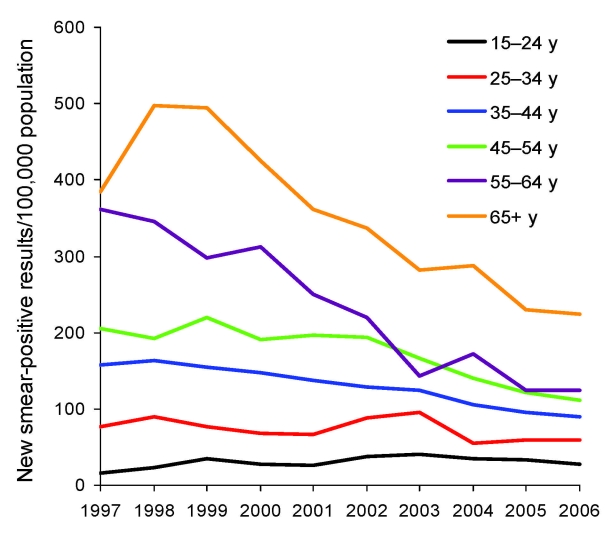
Trends in case notification rates for patients with new smear-positive tuberculosis, by age, Vietnam, 1997–2006. The annual percentage changes were +4.8% for persons 15–24 years of age, –3.3% for those 25–34 years of age, –6.1% for those 35–44 years of age, –6.5% for those 45–54 years of age, –11.5% for those 55–64 years of age, and –7.8% for those >65 years of age.

During 1997–2006, CNRs of new smear-positive TB in the study sites decreased by 5.1% per year (95% confidence interval [CI] 4.4%–5.9%), to a lesser extent for men (4.3%, 95% CI 3.4%–5.2%) than for women (7.1%, 95% CI 6.3%–9.1%). Except for patients 15–24 years of age, these decreasing trends were observed for both sexes (Figure 2) and age groups (Figure 3).

During 2003–2006, a total of 2,337 new smear-positive TB patients were registered for treatment in the 3 participating districts; 2,249 (96%) of these patients met inclusion criteria. We excluded 31 patients who did not have data collection forms and 8 other patients whose sputum samples were lost. Patients excluded for this reason did not differ by age or sex from the other patients. A total of 2,210 (94.9%) culture results were available. Of these results, 84 were negative and 29 grew nontuberculous mycobacteria. The remaining 2,097 isolates (89.7%) were genotyped (Table 1).

**Table 1:** Distribution of *Mycobacterium tuberculosis* genotypes among patients, Vietnam, 2003–2006

Characteristic	No. patients	Genotype							
		Beijing			Vietnam			Other	
		No. (%) patients	p value*		No. (%) patients	p value*		No. (%) patients	p value*
Total	2,097	681 (32.5)			1,063 (50.7)			353 (16.8)	
Year									
2003	369	115 (31.2)	0.396		190 (51.5)	0.835		64 (17.3)	0.793
2004	628	207 (33.0)			319 (50.8)			102 (16.2)	
2005	550	192 (34.9)			270 (49.1)			88 (16.0)	
2006	550	167 (30.4)			284 (51.6)			99 (18.0)	
Sex									
M	1,576	494 (31.3)	0.051		826 (52.4)	0.006		256 (16.2)	0.197
F	521	187 (35.9)			237 (45.5)			97 (18.6)	
Age group, y									
15–24	177	93 (52.5)	<0.001		52 (29.4)	<0.001		32 (18.1)	0.854
25–34	277	98 (35.4)			130 (46.9)			49 (17.7)	
35–44	426	137 (32.2)			216 (50.7)			73 (17.1)	
45–54	380	120 (31.6)			205 (53.9)			55 (14.5)	
55–64	266	92 (34.6)			128 (48.1)			46 (17.3)	
>65	571	141 (24.7)			332 (58.1)			98 (17.2)	

*By χ^2^ test for comparison of proportions of each genotype by year, sex, and age.

Of these 2,097 isolates, 682 (32.5%) were of the Beijing genotype, 1,063 (50.7%) were of the Vietnam EAI-VNM genotype, and 353 (16.8%) were of other genotypes. There were no differences in age and sex among the patients from whom the isolates were obtained and subjected to genotyping and all remaining patients registered during the study period. The proportion of isolates with a Beijing genotype was significantly higher for patients 15–24 years of age (52.5%) than for patients of other ages (30.6%; p<0.001). The proportion of isolates with the Vietnam genotype was higher among men and among patients >24 years of age; no differences were found for the other genotypes when combined into 1 group (Table 1). No time trends were evident after stratification of the data by age group or sex.

Because the proportion of genotypes other than Beijing and Vietnam was low and was composed of a heterogeneous group, we limited analyses of CNRs by genotype to the isolates of the Vietnam and Beijing genotypes. The age groups 25–34, 35–44, 45–54, and 55–64 years were grouped together because we found no variation in genotype distribution within these subgroups (Table 1).

During 2003–2006, the overall CNR for new smear-positive TB decreased by an average of –4.3% (95% CI –8.0% to –0.3%) per year. Decreasing trends were also seen for patients 25–64 years of age (–5.1% per year, 95% CI –9.7% to –0.2%) and >65 years of age (–8.0% per year, 95% CI –14.8% to –0.7%). For patients 15–24 years of age, CNRs showed an average annual increase of +5.2% (95% CI –8.4%–20.9%). Rates for women showed an average decrease of –7.9% per year (95% CI –15.2% to –0.2%) and for men, an average decrease of –3.0% per year (95% CI –7.3%–1.6%).

**Figure 4:**
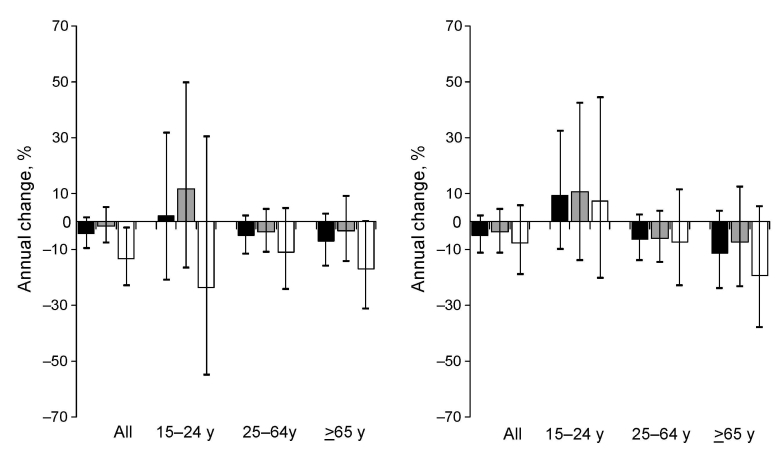
Average annual percentage changes in case notification rates for patients with new smear-positive tuberculosis by age and sex, for the Vietnam genotype (A) and the Beijing genotype (B), Vietnam, 2003–2006. Black columns, total; gray columns, male patients; white columns, female patients. Error bars indicate 95% confidence intervals.

Overall trends in CNRs were similar for patients infected with the Beijing and with the Vietnam genotypes (decreases of –5.9% and –4.4% per year, respectively) (Figure 4). Decreasing trends were observed for middle-age and elderly persons (–6.3% and –5.0% per year for patients 24–64 years of age and –11.3% and –7.1% per year for patients >65 years of age).

However, for patients 15–24 years of age, CNRs increased for patients infected with either genotype. This increase was higher for those with the Beijing genotype (9.2%) than for those with the Vietnam genotype (2.0%), but this difference was not statistically significant (p = 0.860). This difference could be explained by a divergent trend among women: a 7.3% increase per year for those with the Beijing genotype (95% CI –20.2%–44.3%) versus a –13.6% decrease for those with the Vietnam genotype (95% CI –55.1% to –3.5%, p = 0.360) (Figure 4).

**Figure 5:**
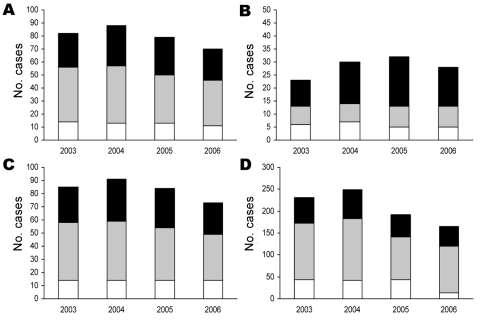
Number of new smear-positive tuberculosis cases, by mycobacterial genotype and patient age, Vietnam, 2003–2006. A), All patients; B) patients 15–24 years of age; C) patients 25–64 years of age; D) patients >65 years of age. White columns, other genotypes; gray columns, Vietnam genotype; black columns, Beijing genotype.

Closer inspection of CNRs during 2003–2006 showed an increase, followed by a decrease, which was consistent across age groups but with different patterns over time (Figure 5). The distribution of genotypes over time differed by age group. After correction for differences in absolute changes between years, the proportion of variation that was caused by the Vietnam genotype was ≈50% for patients >25 years of age but only 3% for patients 15–24 years of age (Figure 5, Table 2). The proportion of variation caused by the Beijing genotype was 85% for patients 15–24 years of age compared with 53% for patients 25–64 years of age and 23% for patients >65 years of age. Differences between the Beijing genotype and remaining genotypes within the youngest and oldest age groups and differences in genotype distribution between the youngest and the oldest age groups were statistically significant (Table 2).

**Table 2:** Annual change in tuberculosis case notification rates, by genotype, Vietnam, 2003–2006*

Age group, y	Absolute change in case rates	Proportion of change caused by				p value			
		Beijing genotype infections	Vietnam genotype infections	Other genotype infections		Within age groups			Among age groups
						Beijing genotype†	Vietnam genotype‡		Beijing genotype§
All	8.0	0.45	0.44	0.11		<0.001	<0.001		
15–24	4.3	0.85	0.03	0.12		<0.001	<0.001		<0.001
25–64	8.0	0.53	0.47	0.00		0.818	0.907		0.818
>65	34.0	0.23	0.56	0.21		0.001	<0.05		0.001

*Per 100,000 population. Average weighted for difference in absolute changes. †By χ^2^ test for comparison between Beijing and remaining genotypes in the same age group. ‡By χ^2^ test for comparison between Vietnam and remaining genotypes in the same age group. §By χ^2^ test for comparison between each age group with the remaining age groups; Beijing vs. all remaining genotypes.

## Discussion

During 1997–2006, CNRs for new smear-positive TB for the study site decreased by 4.3% per year likely due to introduction of the DOTS strategy in 1994. As in other studies in Vietnam (*9**,**10*)*,* we found an underlying pattern of increasing CNRs for young adults, which partially compensated for strongly decreasing CNRs for other age groups.

In the study area, where the Beijing genotype is associated with young age and female sex (*13*), we observed no effect of variations in genotype distribution on the CNRs for all ages and sexes combined. For patients >25 years of age, decreasing trends were observed for the Beijing and Vietnam genotypes. There was also no overall difference in CNRs between men and women. However, for patients 15–24 years of age, an increasing trend for both major genotypes was observed. This trend was stronger for the Beijing genotype than for the Vietnam genotype, although the difference was not significant. This difference was most apparent among women in this age group.

Increases in CNRs among young adults are generally considered to reflect recent increases in transmission (*29*). Therefore, our findings may suggest that the Vietnam genotype is being replaced by the Beijing genotype at the population level in rural Vietnam. The lack of an association between the genotype and the trend in CNRs could then reflect random error or a too-short observation period (4 years). Larger studies of longer duration may be needed to determine whether such an association exists.

The increase in CNRs of new smear-positive TB for persons 15–24 years of age may also be explained by development of the HIV epidemic. HIV increases the risk for progression of *M. tuberculosis* infection to TB and probably increases susceptibility to infection (*1*). In sub-Saharan Africa, 9% of all new TB cases in persons 15–49 years of age were attributable to HIV infection (*8*). In Ho Chi Minh City, the HIV prevalence among TB patients during 1997–2002 increased exponentially from 1.5% to 9% (*11*). This increase in CNRs of new smear-positive TB in patients 15–34 years of age was attributable to HIV, although HIV could not explain the lack of an expected decrease. In our study, HIV data for TB patients were not obtained. The estimated prevalence of HIV infection among persons 15–49 years of age in Tien Giang, Vietnam, in 2005 was much lower than in Ho Chi Minh City (0.5%) (*30*), similar to the national average. The estimated HIV prevalence among TB patients in 30 sentinel provinces was 4.8% in 2004 (D.H. Thanh, Vietnam National Tuberculosis Programme, unpub. data). Thus, it is unlikely that HIV played a role in the observed associations. Furthermore, no association has been found between HIV infection status and isolation of Beijing genotype from pulmonary TB patients elsewhere (*31*).

However, we have alternative explanations for our results. We found that among young adults, nearly all of the variation in CNRs between the years of collection was attributable to variations in CNRs caused by the Beijing genotype; this variation was less apparent for persons in older age groups. This finding suggests that Beijing strains circulate more abundantly among young adults as a consequence of high transmission rates within this age group. Alternatively, the observed fluctuations in numbers of cases caused by Beijing strains may reflect importation from urban areas. Although we did not collect data from these areas, young adults in Tien Giang often travel (70 km) to Ho Chi Minh City for school or work. This explanation is supported by the following findings: the prevalence of infections with the Beijing genotype among persons with TB in this city was higher than the prevalence of comparable patients in rural districts (*13**,**21*) and our earlier result that infections with the Beijing genotype in Tien Giang were more common in patients living along the main road to Ho Chi Minh City (*13*).

High transmission rates among young adults, particularly in urban areas, may increase emergence of Beijing strain TB infections in Vietnam. Recent data from The Gambia suggest that Beijing strain infections do not show increased secondary attack rates but have shorter incubation periods than other genotypes (*32*). In settings with high transmission rates, such strains may be preferentially selected. Even if their risk for transmission as such is not increased, their faster progression to TB and infectiousness will give them a selective advantage. This hypothesis may also explain the association between the Beijing genotype and a history of imprisonment (e.g., in the former Soviet Union) (*19**,**33*).

To test this hypothesis, more studies of genotype-specific variations in incubation period and variations in genotype between high-transmission and low-transmission settings are needed. Different sublineages of the Beijing genotype may have different pathogenic characteristics (*34*,*35*). Data from Vietnam suggest that the more recently evolved typical Beijing strains have a higher propensity to evade immunity from *M*. *bovis* BCG vaccination (*36*). In addition, studies among immigrant populations in the United States, South Africa, and Canada have suggested that the transmission propensity of different genotypes and of different sublineages of the Beijing genotype is dependent on the host population (*19**,**33**,**35**,**37**–**39*). Such studies (*19*,*33*) should enable analyses of different sublineages of the Beijing genotype and involve non-East Asian populations.

The association of the Beijing genotype with young adults could threaten the effectiveness of the DOTS strategy in Vietnam and elsewhere. However, this possibility is unlikely because in our study, although 25% of the elderly patients were infected by Beijing strains, overall CNRs of TB caused by Beijing strains still decreased. Conversely, associations have been found in some studies, including one in the same study area, between the Beijing genotype and drug resistance, particularly for multidrug-resistant TB (MDR TB) (*13**,**17**,**18**,**21**,**31*). Although until 2001 the prevalence of MDR TB among new patients with smear-positive TB in the study area was relatively low and not increasing (*40*), the effects of genotype-associated risks for increased acquisition or transmission of MDR TB may threaten the effectiveness of long-term TB control.

Our study has several other limitations. First, our data were limited to new patients with smear-positive pulmonary TB and may not be representative of patients with other types of TB, including previously treated patients. In our study area, the Beijing genotype was more prevalent in previously treated patients than in new patients (*13*), which suggested that a stronger effect of genotype on TB trends might be observed if previously treated patients were included in the analyses. Second, we did not include patients who were treated in the private health sector. We collected no data on the proportion of patients treated in the private sector, but local health authorities estimate the proportion of these patients to be <10%. Third, we did not include children <15 years of age because of ethical constraints and because their number was expected to be small. In Vietnam, children account for no more than 0.2% of notified smear-positive TB cases (National Tuberculosis Program, unpub. data). However, pediatric TB may be underdiagnosed if the only test for diagnosis is microscopic examination of sputum smears.

Our data do not prove or refute that the increase of TB in young adults in rural Vietnam is related to emergence of the Beijing genotype. However, they do suggest that the association between the Beijing genotype and young age reflects importation of Beijing strain infections from urban areas into rural areas. This importation may be linked to or driven by high rates of transmission among young adults.
